# The Effect of Axial Length on Macular Vascular Density in Eyes with High Myopia

**DOI:** 10.22336/rjo.2025.15

**Published:** 2025

**Authors:** Mustafa Kayabaşi, Seher Köksaldi, Neslihan Demirel, Ali Osman Saatci

**Affiliations:** 1 Department of Ophthalmology, Mus State Hospital, Mus, Turkey; 2 Department of Ophthalmology, Agri Ibrahim Cecen University, Agri, Turkey; 3 Department of Statistics, Dokuz Eylul University, Izmir, Turkey; 4 Department of Ophthalmology, Dokuz Eylul University, Izmir, Turkey

**Keywords:** degenerative myopia, foveal avascular zone, high myopia, optical coherence tomography angiography, vascular density, AL = Axial length, BCVA = Best corrected visual acuity, BM = Bruch’s membrane, CC = Choriocapillaris, CMT = Central macular thickness, CFI = Color fundus image, DCP = Deep capillary plexus, EDI = Enhanced depth imaging, FAZ = Foveal avascular zone, HM = High myopia, ILM = Internal limiting membrane, META-PM = Meta-Analysis for Pathologic Myopia, MNV = Macular neovascularization, OCT = Optical coherence tomography, OCTA = Optical coherence tomography angiography, OuR = Outer retina, PS = Posterior staphyloma, RPE = Retinal pigment epithelium, SCT = Subfoveal choroidal thickness, SCP = Superficial capillary plexus, SE = Spherical equivalent, VD = Vascular density

## Abstract

**Objective:**

To evaluate the relationship between optical coherence tomography angiography (OCTA) findings and axial length (AL) in eyes with high myopia.

**Materials and Methods:**

A total of 122 eyes from 78 patients were included. Seventy-five eyes with an AL ranging between 26.00 and 27.49 mm comprised Group 1, and 47 with an AL of ≥ 27.50 mm comprised Group 2. Spectral-domain OCT was performed to measure the central macular thickness, subfoveal choroidal thickness (SCT) and swept-source OCTA was utilized to obtain the data on foveal avascular zone (FAZ) and vascular density (VD) values at the superficial and deep capillary plexuses (SCP and DCP), outer retina (OuR), and choriocapillaris (CC) segments.

**Results:**

While no significant differences were found in terms of the mean superficial-FAZ and deep-FAZ areas (*p*=0.284 and *p*=0.952, respectively), there were significant differences between the groups in terms of the mean foveal VD in the SCP (*p*=0.001), the mean total VD (*p*=0.045) and foveal VD in the DCP (*p*<0.001), the mean foveal VD (*p*=0.019) and superior parafoveal VD in the OuR (*p*=0.008), the mean total (*p*=0.005), temporal parafoveal (*p*=0.034), inferior parafoveal (*p*=0.029), and nasal parafoveal VDs in the CC segments (*p*=0.005).

**Discussion:**

The findings of the present study highlight the complex interplay between axial elongation and retinal microvasculature, suggesting that factors beyond mechanical stretching may contribute to these alterations. The variability in the existing literature on this topic arises from inconsistencies in the definition of high myopia, the use of different OCTA devices, and heterogeneous study populations. By including eyes with myopic maculopathy and employing axial length-based classification, this study provides a broad representation of high myopia. However, its retrospective design, single-center setting, and monoracial cohort represent limitations. Future large-scale, prospective studies involving diverse populations are needed to elucidate further the pathophysiology of high myopia and its impact on retinal and choroidal microcirculation.

**Conclusions:**

Our study revealed that high-myopic eyes with longer ALs exhibited increased total VD in the DCP and increased foveal VD in the SCP, DCP, and OuR segments, while they showed decreased total VD and temporal, inferior, and nasal parafoveal VDs in the CC segment compared to high-myopic eyes with shorter ALs.

## Introduction

Due to its increasing prevalence over the past three decades, high myopia (HM) has become a significant public health concern. However, determining the prevalence of HM is challenging due to the lack of a standardized definition for HM. Nevertheless, reports indicate a global prevalence of 4%, with projections anticipating a rise to 9.8% by 2050 [1,2].

Several sight-threatening pathological changes, such as chorioretinal atrophy, lacquer crack, myopic maculopathy, posterior staphyloma (PS), macular neovascularization (MNV), peripheral retinal degeneration, retinal breaks, and rhegmatogenous retinal detachment, may develop in eyes with HM [3-5]. Recent publications have indicated that these changes might be associated with vascular alterations at the retinal or choroidal level [6-8].

High myopia-related macular complications may lead to severe vision loss. Therefore, ophthalmologists must identify any macular vascular changes in high myopic eyes. Optical Coherence Tomography Angiography (OCTA) technology has revolutionized non-invasive imaging by capturing consecutive OCT images and utilizing signals from red blood cell movement to visualize the retina, choroid, and optic nerve microvascular structures without the need for contrast agents. Its segmentation capability enables us to obtain quantitative data, including foveal avascular zone (FAZ) width and vascular density (VD) values at the levels of retinal plexuses and choroid [9,10]. Thus, these measurements provide new insights into understanding the underlying mechanisms of various retinal and choroidal diseases, including diabetic retinopathy, retinal vascular occlusion, age-related macular degeneration, and HM [5,11,12].

In this study, we aim to present our clinical observations in eyes with HM using swept-source OCTA and assess the relationship between chorioretinal microvascular changes and axial length (AL).

## Materials and Methods

This cross-sectional study was conducted in accordance with the principles outlined in the Declaration of Helsinki and received approval from the local ethics committee at Dokuz Eylul University (Approval ID: 2023/06-23). Patient files and concurrent imaging data (spectral-domain OCT images, swept-source OCTA images, and color fundus images—CFI) of patients diagnosed with HM at the Department of Ophthalmology, Dokuz Eylul University, between January 2019 and January 2023, were retrospectively analyzed.

The inclusion criteria were determined as having an AL ≥ 26.00 mm and the availability of OCT, OCTA, and CFI obtained at the same visit. Eyes with any coexisting vitreous abnormalities (e.g., asteroid hyalosis), significant lens opacity (cataract grade ≥ 3 according to the Emery-Little classification) [13] that might have affected the OCT and OCTA image quality, a previous history of any ocular surgeries (refractive surgery, vitreoretinal surgery, cataract surgery, glaucoma surgery), poor-quality OCT images (signal-to-noise ratio < 18 dB), poor-quality OCTA images (< 40), or segmentation errors on OCTA were excluded. The presence or absence of anterior retinal changes was not considered for the inclusion or exclusion of the eyes, as the primary objective was to evaluate chorioretinal microvascular changes at the posterior pole using OCTA. Additionally, patients under 18 years of age, patients with any ocular or systemic diseases except for HM, patients with a history of ocular trauma, and patients with a history of systemic drug usage were also excluded from the study.

Demographic data, refractive status detected with Nidek TonoRef II auto-refracto-keratometer (NIDEK Co, Ltd, Japan), Snellen best-corrected visual acuity (BCVA), and AL measurements obtained with IOL Master 500 optic biometry (Carl Zeiss Meditec, Jena, Germany) were taken from the medical records of the patients. The spherical equivalent (SE) was calculated by adding half of the cylindrical refractive error to the spherical refractive error. The decimal BCVA was converted to logMAR for statistical analysis.

The Meta-Analysis for Pathologic Myopia (META-PM) category (category 0: normal fundus, category 1: tigroid fundus, category 2: diffuse chorioretinal atrophy, category 3: patchy chorioretinal atrophy, and category 4: macular atrophy) [14] was determined in all eyes based on the 45-degree CFIs centered on the fovea obtained with the Zeiss VISUCAM 500 (Carl Zeiss Meditec, Jena, Germany).

**Fig. 1:**
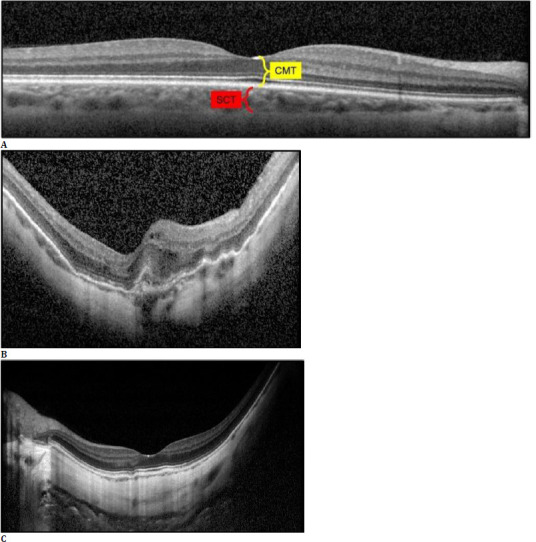
**A**. The measurements of the central macular thickness (CMT) (indicated in yellow) and subfoveal choroidal thickness (SCT) (shown in red) are illustrated on a transfoveal 7-mm horizontal spectral-domain optical coherence tomographic (OCT) section of the right eye of a 34-year-old male with an axial length (AL) of 27.40 mm; **B**. An active macular neovascularization is observed as a hyperreflective membrane above the retinal pigment epithelium, located within the outer retinal layers in association with intraretinal fluid on the transfoveal 7-mm horizontal spectral-domain OCT section of the right eye of a 63-year-old female with an AL of 28.54 mm; **C**. A posterior staphyloma is observed as an outward protrusion of sclera with a curvature radius smaller than the curvature radius of the adjacent sclera on the transfoveal 9-mm enhanced depth imaging OCT of a 33-year-old male with an AL of 29.31 mm

Central macular thickness (CMT) was measured as the closest distance between the internal limiting membrane (ILM) and Bruch’s membrane (BM) by using a built-in software [15] (**Fig. 1 A**). The presence of an MNV was determined as the presence of a hyperreflective membrane above the retinal pigment epithelium (RPE), located within the outer retinal layers on the transfoveal 7-mm horizontal spectral-domain OCT section (Heidelberg Spectralis, Heidelberg Engineering, Heidelberg, Germany) [4] (**Fig. 1 B**). Macular neovascularization accompanied by hemorrhage or intraretinal/subretinal fluid were categorized as active MNV, while those not accompanied by these findings were recorded as inactive MNV.

Subfoveal choroidal thickness (SCT) was measured as the closest distance between the hyperreflective outer border of the RPE and the choroid-sclera junction by using a built-in software [16] (**Fig. 1 A**). PS was defined as an outward protrusion of sclera with a curvature radius smaller than the curvature radius of the adjacent sclera on the transfoveal 9-mm enhanced depth imaging (EDI) OCT (Heidelberg Spectralis, Heidelberg Engineering, Heidelberg, Germany) [17] (**Fig. 1 C**).

**Fig. 2:**
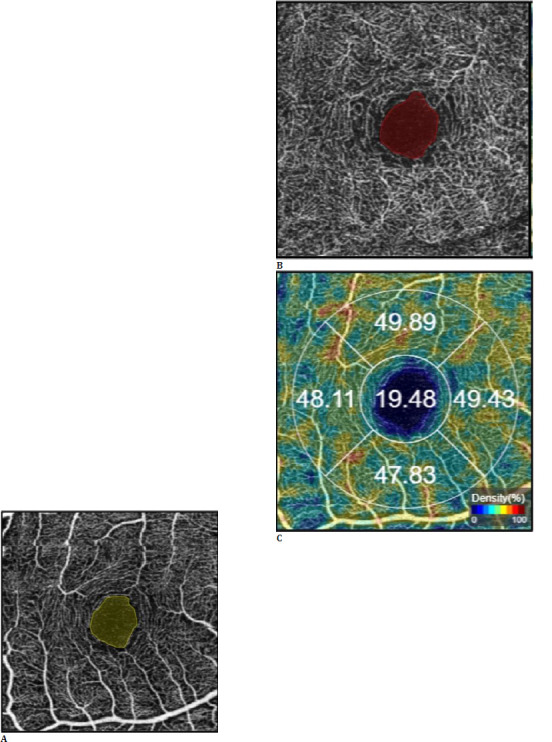
Illustration of the 3 x 3 mm optical coherence tomography angiography findings of the right eye of a 26-year-old female with an axial length of 27.43 mm. The foveal avascular zone area was manually measured in the layers of the superficial capillary plexus (**A**) - yellow area and the deep capillary plexus (**B**) - red area. Vascular density (VD) values were recorded from the density map as the foveal (19.48%) and superior (49.89%), temporal (48.11%), inferior (47.83%), and nasal parafoveal (49.43%) VDs (**C**)

Optical coherence tomography angiography images were acquired using the angio retina scanning mode of the Topcon DRI OCT Triton device (Topcon Inc., Tokyo, Japan). The scanning area used in this study was 3 × 3 mm, centered on the fovea. Automatic segmentation lines were used to determine the superficial capillary plexus (SCP), deep capillary plexus (DCP), outer retina (OuR), and choriocapillaris (CC) layers. The segment extending from 2.6 μm below the ILM to 15.6 μm below the junction of the inner plexiform and inner nuclear layers was defined as the SCP. The segment between 15.6 and 70.2 μm below the intersection of the inner plexiform and inner nuclear layers was referred to as DCP. The segment extending from 70.2 μm below the junction of the inner plexiform and inner nuclear layers to the BM was segmented as the outer retina (OuR) and further segmented as the CC up to 10.4 μm below the BM. The FAZ areas of the SCP and DCP segments were manually measured using the device’s built-in software and recorded as the superficial FAZ area (**Fig. 2 A**) and deep FAZ area (**Fig. 2 B**), respectively. The FAZ areas assessed from the OCTA images were reanalyzed to correct for the magnification in the study eyes and adjusted according to AL using Bennett’s formula [18], which is considered a more accurate correction factor than the method using keratometry [19]. Vascular density data were automatically recorded within a central area of 1 mm diameter for the SCP, DCP, OuR, and CC segments, as well as in areas 1 mm superior, nasal, inferior, and temporal to the outermost boundary of this area [20] (**Fig. 2 C**). The parafoveal VD in a segment was calculated as the arithmetic mean of VDs in the superior, temporal, inferior, and nasal parafoveal quadrants. The total VD in a segment was determined by calculating the arithmetic mean of VDs in both the fovea and parafoveal quadrants.

The eyes enrolled in the study were divided into two groups: Group 1 consisted of eyes with an axial length (AL) ranging from 26.00 mm to 27.49 mm, while Group 2 consisted of eyes with an AL of 27.50 mm or above. The data obtained was compared between these two groups.

### Statistical Analysis

Statistical analyses were performed using SPSS Statistics Version 28 (IBM, Armonk, New York, USA). Descriptive statistics summarized the data, with categorical variables presented as counts and percentages and quantitative variables reported as means ± standard deviations. The normality of data distribution was assessed via the Kolmogorov-Smirnov and Shapiro-Wilk tests. A parametric independent sample t-test was used to compare normally distributed continuous variables. Non-normally distributed variables were compared using the nonparametric Mann-Whitney U test. Categorical variables underwent analysis through the chi-square test (with or without Yates continuity correction) and Fisher’s exact test whenever necessary. Spearman's correlation was used to assess the relationship between the OCTA parameters of the macular microvasculature and AL. A *p*-value below 0.05 was considered statistically significant.

## Results

After evaluating 156 eyes from 96 patients with HM, 34 eyes were excluded due to segmentation errors on OCTA, resulting in a total of 122 eyes (68 right eyes, 54 left eyes) from 78 patients. 48 (61.5%) were female and 30 (38.5%) male. The mean age of the whole cohort was 43.88 ± 16.20 years (range: 18-84 years). The eyes of 44 patients (56.4%) were considered. Group 1 comprised 75 eyes from 52 patients, while Group 2 consisted of 47 eyes from 32 patients. The two groups did not exhibit statistically significant differences in terms of sex (corrected chi-square test, *p* = 0.974). Although the mean age of Group 2 was higher than that of Group 1, the difference was not statistically significant (Mann-Whitney U test, *p* = 0.203) (**Table 1**).

**Table 1:** Clinical characteristics of the study patients and eyes

	Group 1	Group 2	*p*-value
**Number of patients/eyes**, n	52 / 75	32 / 47	-
**Age**, years	41.27 ± 16.54	46.06 ± 15.86	0.203^a^
**Sex**, M / F	21 / 31	12 / 20	0.974^b^
**Mean SE value**, diopters	-9.09 ± 3.40	-13.80 ± 3.90	**<0.001^a^**
**AL**, mm	26.55 ± 0.46	29.67 ± 2.21	**<0.001^a^**
**BCVA**, logMAR	0.18 ± 0.21	0.50 ± 0.42	**<0.001^a^**
**Mean CMT**, μm	233.79 ± 35.87	247.77 ± 47.67	0.088^c^
**Presence of MNV**, n (%)	2 (2.6)	10 (21.3)	**0.001^d^**
**Mean SCT**, μm	211.89 ± 73.73	121.83 ± 91.98	**<0.001^a^**
**Presence of PS**, n (%)	3 (4)	21 (44.7)	**<0.001^d^**

AL = axial length, BCVA = best corrected visual acuity, CMT = central macular thickness, F = female, M = male, MNV = macular neovascularization, PS = posterior staphyloma, SCT = subfoveal choroidal thickness, SE = spherical equivalent

a Mann-Whitney U test

b Corrected chi-square test

c Independent samples t-test

d Fisher’s exact test

There were statistically significant differences between the two groups in terms of the mean SE value, AL, and BCVA (Mann-Whitney U test, *p* < 0.001 for all), as expected (**Table 1**).

Eyes were classified according to the META-PM classification. Nine eyes (12%) were in category 0, 46 eyes (61.3%) in category 1, and 20 eyes (26.7%) in category 2 in Group 1. Two eyes (4.3%) were in category 0, 16 eyes (34%) in category 1, 14 eyes (29.8%) in category 2, seven eyes (14.9%) in category 3, and eight eyes (17%) in category 4 in Group 2. The chi-square test revealed a significant difference between the two groups (*p* < 0.001).

Even though the mean CMT was slightly thinner in Group 1 than in Group 2 (233.79 ± 35.87 μm and 247.77 ± 47.67 μm, respectively), the difference was not statistically significant (independent samples t-test, *p* = 0.088). Two eyes (2.6%) in Group 1 had MNV (one active, one inactive), whereas 10 eyes (21.3%) in Group 2 exhibited MNV (four active, six inactive) on OCT (Fisher’s exact test, *p* = 0.001) (**Table 1**).

The mean SCT was 211.89 ± 73.73 μm (range: 57-391 μm) and 121.83 ± 91.98 μm (range: 16-433 μm) for Group 1 and Group 2, respectively (independent samples t-test, *p* < 0.001). Posterior staphyloma was detected in three eyes (4%) in Group 1 and 21 eyes (44.7%) in Group 2 on EDI-OCT (Fisher’s exact test, *p* < 0.001) (**Table 1**).

Even though Group 1 had a larger superficial-FAZ area compared to Group 2 (253.87 ± 107.11 μm^2^ and 227.43 ± 76.48 μm^2^, respectively), the difference was not statistically significant (*p* = 0.284 with Mann-Whitney U test). There was also no statistically significant difference between the groups in terms of the mean deep-FAZ area (Mann-Whitney U test, *p* = 0.952), despite Group 1 having a slightly larger deep FAZ area (261.18 ± 103.35 μm^2^) compared to Group 2 (256.69 ± 89.68 μm^2^).

There were no statistically significant differences in the mean total VD between the two groups in the SCP (Mann-Whitney U test, *p* = 0.427) and OuR segments (independent samples t-test, *p* = 0.301). Nevertheless, in the DCP segment, the mean total VD was 45.40 ± 2.08% for Group 1 and 46.37 ± 2.62% for Group 2, revealing a significant difference (Mann-Whitney U test, *p* = 0.045). Additionally, in the CC segment, Group 1 exhibited a significantly higher mean total VD than Group 2 (53.20 ± 1.41% vs. 52.62 ± 1.36%, respectively; p = 0.005, independent samples t-test). Upon the mean VD values compared by quadrants, there were statistically significant differences between Group 1 and Group 2 in terms of the mean foveal VD in the SCP segment (21.23 ± 4.31% and 23.41 ± 3.46%, respectively; *p* = 0.001 with Mann-Whitney U test), the mean foveal VD in the DCP segment (20.54 ± 5.22% and 25.05 ± 6.35%, respectively; *p* < 0.001 with Mann-Whitney U test), the mean foveal VD (40.29 ± 4.66% and 44.56 ± 9.42%, respectively; *p* = 0.019 with Mann-Whitney U test) and the superior parafoveal VD in the OuR segment (53.70 ± 2.20% and 51.93 ± 4.70%, respectively; *p* = 0.008 with Mann-Whitney U test), the mean temporal parafoveal VD (54.62 ± 2.53% and 53.55 ± 2.85%, respectively; *p* = 0.034 with independent samples t-test), the mean inferior parafoveal VD (53.15 ± 2.33% and 51.83 ± 3.64%, respectively; *p* = 0.029 with independent samples t-test), and the mean nasal parafoveal VD in the CC segment (53.49 ± 2.56% and 52.14 ± 2.46%, respectively; *p* = 0.005 with independent samples t-test). There were no statistically significant differences among the VD values of the other quadrants between the two groups (*p* > 0.05). The optical coherence tomography angiography findings of the two groups are detailed in **Table 2**.

**Table 2:** Optical coherence tomography angiography findings of the study eyes

	Group 1 (n=75)	Group 2 (n=47)	*p*-value
**Superficial-FAZ**, μm^2^	253.87 ± 107.11	227.43 ± 76.48	0.284^a^
**Deep-FAZ**, μm^2^	261.18 ± 103.35	256.69 ± 89.68	0.952^a^
**Superficial capillary plexus**			
Total VD, %	43.95 ± 1.95	44.00 ± 2.98	0.427^a^
Foveal VD, %	21.23 ± 4.31	23.41 ± 3.46	**0.001^a^**
Parafoveal VD, %	49.62 ± 2.46	49.15 ± 3.58	0.074^a^
Superior parafoveal VD, %	51.33 ± 4.12	50.18 ± 5.35	0.327^a^
Temporal parafoveal VD, %	48.34 ± 3.61	48.80 ± 4.65	0.535^b^
Inferior parafoveal VD, %	50.96 ± 4.47	51.30 ± 6.92	0.768^b^
Nasal parafoveal VD, %	47.86 ± 4.06	46.34 ± 5.30	0.075^b^
**Deep capillary plexus**			
Total VD, %	45.40 ± 2.08	46.37 ± 2.62	**0.045^b^**
Foveal VD, %	20.54 ± 5.22	25.05 ± 6.35	**<0.001^a^**
Parafoveal VD, %	51.75 ± 2.61	51.71 ± 3.73	0.948^b^
Superior parafoveal VD, %	52.03 ± 3.86	50.87 ± 6.62	0.280^b^
Temporal parafoveal VD, %	48.62 ± 3.52	50.27 ± 6.85	0.606^a^
Inferior parafoveal VD, %	54.16 ± 4.92	54.42 ± 7.59	0.827^a^
Nasal parafoveal VD, %	52.18 ± 4.62	51.26 ± 8.26	0.491^b^
**Outer retina**			
Total VD, %	49.97 ± 1.69	50.38 ± 2.37	0.301^b^
Foveal VD, %	40.29 ± 4.66	44.56 ± 9.42	**0.019^a^**
Parafoveal VD, %	52.39 ± 1.50	51.84 ± 2.10	0.122^b^
Superior parafoveal VD, %	53.70 ± 2.20	51.93 ± 4.70	**0.008^a^**
Temporal parafoveal VD, %	52.22 ± 3.09	52.52 ± 3.91	0.636^b^
Inferior parafoveal VD, %	52.25 ± 3.05	52.07 ± 6.04	0.996^a^
Nasal parafoveal VD, %	51.39 ± 3.16	50.84 ± 3.88	0.638^a^
**Choriocapillaris**			
Total VD, %	53.20 ± 1.41	52.62 ± 1.36	**0.005^a^**
Foveal VD, %	51.22 ± 4.42	52.57 ± 5.41	0.134^a^
Parafoveal VD, %	53.69 ± 1.29	52.63 ± 1.56	**<0.001^a^**
Superior parafoveal VD, %	53.52 ± 2.16	52.98 ± 3.38	0.199^a^
Temporal parafoveal VD, %	54.62 ± 2.53	53.55 ± 2.85	**0.034^b^**
Inferior parafoveal VD, %	53.15 ± 2.33	51.83 ± 3.64	**0.029^b^**
Nasal parafoveal VD, %	53.49 ± 2.56	52.14 ± 2.46	**0.005^b^**

FAZ = foveal avascular zone, VD = vascular density

a Mann-Whitney U test

b Independent samples t-test

No significant correlation was found between AL and the superficial FAZ (*r* = -0.125, *p* = 0.183) and the deep FAZ areas (*r* = -0.031, *p* = 0.741). The total VD in the DCP and the foveal VD in the SCP, DCP, and OuR segments were positively correlated with AL (*r* = 0.217 and *p* = 0.016; *r* = 0.254 and *p* = 0.005; *r* = 0.401 and *p* < 0.001; *r* = 0.216 and *p* = 0.017, respectively). Significant negative correlations were found between the total VD together with the temporal, inferior and nasal VDs in the CC segment and AL (*r* = -0.274 and *p* = 0.002; *r* = -0.195 and *p* = 0.032; *r* = -0.215 and *p* = 0.017; *r* = -0.222 and *p* = 0.014, respectively) (**Table 3**).

**Table 3:** Spearman’s correlation analysis between axial length and optical coherence tomography angiography findings

	SCP	DCP	OuR	CC
**FAZ**, *r (p)*	-0.125 (0.183)	-0.031 (0.741)	-	-
**Total VD**, *r (p)*	-0.085 (0.354)	**0.217 (0.016)**	0.125 (0.170)	**-0.274 (0.002)**
**Foveal VD**, *r (p)*	**0.254 (0.005)**	**0.401 (<0.001)**	**0.216 (0.017)**	0.032 (0.731)
**Parafoveal VD**, *r (p)*	-0.168 (0.065)	-0.006 (0.949)	-0.109 (0.233)	**-0.310 (0.001)**
**Superior parafoveal VD**, *r (p)*	-0.054 (0.558)	-0.033 (0.718)	-0.117 (0.198)	-0.043 (0.641)
**Temporal parafoveal VD**, *r (p)*	-0.006 (0.949)	0.045 (0.632)	0.038 (0.674)	**-0.195 (0.032)**
**Inferior parafoveal VD**, *r (p)*	0.014 (0.880)	0.076 (0.403)	0.049 (0.595)	**-0.215 (0.017)**
**Nasal parafoveal VD**, *r (p)*	-0.028 (0.759)	-0.028 (0.759)	0.010 (0.914)	**-0.222 (0.014)**

CC = choriocapillaris, DCP = deep capillary plexus, FAZ = foveal avascular zone, OuR = outer retina, SCP = superficial capillary plexus, VD = vascular density

## Discussion

Our study revealed no significant differences between the groups in terms of the superficial and deep FAZ areas, along with an increase in the total VD in the DCP segment, accompanied by an increased foveal VD in the SCP, DCP, and OuR segments in high myopic eyes with longer ALs. In contrast, these eyes exhibited a decrease in the superior parafoveal VD in the OuR segment, as well as a reduction of the total, temporal parafoveal, inferior parafoveal, and nasal parafoveal VDs in the CC segment compared to the eyes with shorter ALs. In addition, AL exhibited a positive correlation with the total VD in the DCP and the foveal VD in the SCP, DCP, and OuR segments while showing a negative correlation with the total and temporal, inferior, nasal parafoveal VDs in the CC segment.

Optical Coherence Tomography Angiography was used to evaluate retinal and choroidal microvasculature alterations in eyes with HM; however, the results have been inconsistent to date, as reported by several authors (**Table 4, 5**) [6-8, 10, 21-32]. One of the leading causes of these contradictory results was the use of diverse definitions for HM, as there is no established definition. Some authors considered the eyes to be high myopic based on the SE value [33-35], while others used the AL for classification [24,25,36]. Additionally, some employed both criteria [26,37,38]. The heterogeneity of definitions makes it challenging to compare results across different publications. We believe that classification based solely on SE value may not be a suitable approach as HM is a degenerative process that may show progression as well as ongoing possible age-related nuclear sclerosis related to myopic shift. Therefore, we chose AL as the main criterion when defining the HM in our study.

**Table 4:** Previous publications comparing the optical coherence tomography angiography findings among myopic eyes

Author^Ref^	Year	HM Criteria	Number of Eyes	Parameter	Significant Differences
Meng et al. [6]	2022	SE<-6.00 D	54 HM 66 Moderate myopia 40 Mild myopia	FAZ area Whole VD in SCP Superior VD in SCP Temporal VD in SCP Inferior VD in SCP Nasal VD in SCP Foveal VD in SCP Whole VD in DCP Superior VD in DCP Temporal VD in DCP Inferior VD in DCP Nasal VD in DCP Foveal VD in DCP Flow area in OuR Flow area in CC	- HM < Moderate < Mild HM < Moderate < Mild HM < Moderate < Mild HM < Moderate < Mild HM < Moderate < Mild - HM < Moderate < Mild HM < Moderate < Mild - - - - - HM < Moderate < Mild
Jiang et al. [7]	2021	SE<-6.00 D	35 HM 35 Non-HM	Whole VD in SCP Foveal VD in SCP Parafoveal VD in SCP Superior-hemi VD in SCP Inferior-hemi VD in SCP Whole VD in DCP Foveal VD in DCP Parafoveal VD in DCP Superior-hemi VD in DCP Inferior-hemi VD in DCP Whole VD in CC Foveal VD in CC Parafoveal VD in CC Superior-hemi VD in CC Inferior-hemi VD in CC	HM < Non-HM - HM < Non-HM HM < Non-HM HM < Non-HM HM < Non-HM - - HM < Non-HM - - - - - -
Wang et al. [10]	2021	SE≤-6.00 D	30 HM 30 Moderate myopia 30 Mild myopia	Whole VD in SCP Parafoveal VD in SCP Superior VD in SCP Temporal VD in SCP Inferior VD in SCP Nasal VD in SCP Foveal VD in SCP Whole VD in DCP Parafoveal VD in DCP Superior VD in DCP Temporal VD in DCP Inferior VD in DCP Nasal VD in DCP Foveal VD in DCP	HM < Other groups HM < Other groups HM < Other groups HM < Other groups HM < Other groups HM < Other groups - HM < Other groups HM < Other groups HM < Other groups HM < Other groups - HM < Other groups -
Cheng et al. [21]	2022	AL>26.00 mm	69 AL≥28.00 390 AL 27.00-27.99 558 AL 26.00-26.99	Average flow deficit in CC Foveal flow deficit in CC Parafoveal flow deficit in CC Perifoveal flow deficit in CC	(AL ≥ 28.00) group > Other groups - - AL ≥ 28.00) group > Other groups
Yang et al. [22]	2020	SE≤-6.00 D	41 HM 45 Moderate myopia 42 Mild myopia	Superficial-FAZ area Whole VD in SCP Whole VD in DCP	HM < Moderate < Mild HM < Mild HM < Mild
Yao et al. [23]	2022	SE<-6.00 D or AL>26.00 mm	40 HM 58 Moderate myopia 25 Low myopia	FAZ area Whole VD in SCP Foveal VD in SCP Parafoveal VD in SCP Superior para-VD in SCP Temporal para-VD in SCP Inferior para-VD in SCP Nasal para-VD in SCP Perifoveal VD in SCP Superior peri-VD in SCP Temporal peri-VD in SCP Inferior peri-VD in SCP Nasal peri-VD in SCP Whole VD in DCP Foveal VD in DCP Parafoveal VD in DCP Superior para-VD in DCP Temporal para-VD in DCP Inferior para-VD in DCP Nasal para-VD in DCP Perifoveal VD in DCP Superior peri-VD in DCP Temporal peri-VD in DCP Inferior peri-VD in DCP Nasal peri-VD in DCP	HM < Other groups HM < Low - - - - - - - - HM < Moderate < Low - - HM < Low - HM < Other groups HM < Other groups HM < Moderate < Low HM < Other groups HM < Moderate < Low HM < Moderate < Low HM < Moderate < Low HM < Moderate < Low HM < Other groups HM < Other groups

AL = axial length, CC = choriocapillaris, DCP = deep capillary plexus, FAZ = foveal avascular zone, HM = high myopia, OuR = outer retina, PM = pathological myopia, SCP = superficial capillary plexus, SE = spherical equivalent, VD = vascular density

**Table 5:** Previous publications comparing the optical coherence tomography angiography findings between eyes with high myopia and control eyes

Author^Ref^	Year	HM Criteria	Number of Eyes	Parameter	Significant Differences
Yaprak et al. [8]	2021	AL>26.00 mm	35 HM 35 Control	Whole VD in SCP Superior-hemi VD in SCP Inferior-hemi VD in SCP Foveal VD in SCP Parafoveal VD in SCP Perifoveal VD in SCP Whole VD in DCP Superior-hemi VD in DCP Inferior-hemi VD in DCP Foveal VD in DCP Parafoveal VD in DCP Perifoveal VD in DCP	HM < Control HM < Control HM < Control - HM < Control HM < Control HM < Control HM < Control HM < Control - HM < Control HM < Control
Ucak et al. [24]	2020	AL≥26.00 mm	92 HM 80 Control	Superficial-FAZ area Deep-FAZ area Whole VD in SCP Whole VD in DCP	- - HM < Control HM < Control
Min et al. [25]	2020	AL≥26.50 mm	52 HM 52 Control	FAZ area Parafoveal VD in SCP Superior VD in SCP Temporal VD in SCP Inferior VD in SCP Nasal VD in SCP Parafoveal VD in DCP Superior VD in DCP Temporal VD in DCP Inferior VD in DCP Nasal VD in DCP Flow void area in CC	HM > Control HM < Control HM < Control HM < Control HM < Control HM < Control - - - - - -
Ye et al. [26]	2020	SE≤-6.00 D or AL≥26.50 mm	22 PM 48 HM 21 Control	Whole VD in SCP Whole VD in DCP	PM < Control; HM < Control PM < HM < Control
Xu et al. [27]	2021	SE≤-6.00 D and AL≥26.50 mm	64 HM 54 Control	Superficial-FAZ area Superficial-FAZ volume Deep-FAZ area Deep-FAZ volume Total FAZ volume	HM > Control HM > Control HM > Control - -
Su et al. [28]	2020	SE≤-6.00 D or AL≥26.50 mm	25 HM 25 Moderate myopia 25 Control	Whole VD in SCP Whole VD in DCP Flow deficit in CC	HM < Control - HM > Moderate; HM > Control
Wu et al. [29]	2020	SE<-6.00 D	31 HM 31 Control	Superficial-FAZ area Whole VD in SCP Foveal VD in SCP Parafoveal VD in SCP Whole VD in DCP Foveal VD in DCP Parafoveal VD in DCP	HM > Control HM < Control HM < Control HM < Control HM < Control - HM < Control
Lee et al. [30]	2020	AL≥26.00 mm	30 HM 34 Control	Superficial-FAZ area Deep-FAZ area	HM > Control HM > Control
Kocer et al. [31]	2022	AL≥26.00 mm	29 PM 32 HM 37 Control	FAZ area Whole VD in SCP Superior-hemi VD in SCP Inferior-hemi VD in SCP Parafoveal VD in SCP Perifoveal VD in SCP Foveal VD in SCP Whole VD in DCP Superior-hemi VD in DCP Inferior-hemi VD in DCP Parafoveal VD in DCP Perifoveal VD in DCP Foveal VD in DCP	PM < HM < Control PM < HM < Control PM < HM < Control PM < HM < Control PM < HM < Control PM < HM < Control PM < Control; HM < Control PM < HM < Control PM < HM < Control PM < HM < Control PM < HM < Control PM < HM < Control HM < Control
Zhu et al. [32]	2021	AL≥26.00 mm	40 HM 54 Moderate-low 55 Control	Whole VD in SCP Foveal VD in SCP Parafoveal VD in SCP Superior para-VD in SCP Temporal para-VD in SCP Inferior para-VD in SCP Nasal para-VD in SCP Perifoveal VD in SCP Superior peri-VD in SCP Temporal peri-VD in SCP Inferior peri-VD in SCP Nasal peri-VD in SCP Whole VD in DCP Foveal VD in DCP Parafoveal VD in DCP Superior para-VD in DCP Temporal para-VD in DCP Inferior para-VD in DCP Nasal para-VD in DCP Perifoveal VD in DCP Superior peri-VD in DCP Temporal peri-VD in DCP Inferior peri-VD in DCP Nasal peri-VD in DCP	- - - - - - - - - - HM < Control - HM < Other groups - - HM < Control - HM < Other groups HM < Control HM < Control HM < Control HM < Control HM < Control HM < Other groups

AL = axial length, CC = choriocapillaris, DCP = deep capillary plexus, FAZ = foveal avascular zone, HM = high myopia, OuR = outer retina, PM = pathological myopia, SCP = superficial capillary plexus, SE = spherical equivalent, VD = vascular density

While some authors formed a control group from emmetropic eyes for comparison with high myopic eyes [31,32,39], others chose to compare eyes with HM within the myopic group by classifying them according to the SE value as HM, moderate myopia, and low myopia [10,22,23]. The inclusion or exclusion of eyes with pathological changes and myopic maculopathy equal to or higher than the META-PM category 2 was another variable, as alternative classifications were based on the presence or absence of pathological changes, such as HM and PM, as stated by some authors [26,31,35]. In the present study, we did not form an emmetropic control group. We opted not to exclude the eyes with pathological changes such as myopic maculopathy equal to or higher than META-PM category 2, MNV, and/or PS. We included all high myopic eyes whenever high-quality OCT and OCTA images were obtained without segmentation errors. Therefore, we aimed to provide a comprehensive representation of high myopic eyes and successfully applied our findings in daily clinical practice.

Moreover, age was a key determinant in some previous studies on HM, as several authors investigated OCTA findings in children [12,32], others in young adults [6,33,40], and still others in patients with a wide range of ages [24,34,37], similar to our cohort. Thus, age selection might have a role in the inconsistent results among the studies.

Utilizing different OCTA devices was another key factor contributing to the conflicting results. Parameters such as scan rate, wavelength, and the algorithm used to capture signals for erythrocyte movement vary when employing different OCTA devices. Additionally, while specific OCTA devices and software can automatically provide FAZ area and VD values, researchers may need to manually measure or calculate these parameters when using specific OCTA devices. In addition, considering the unique anatomical structure and physiological features of the FAZ, fovea, parafovea, and perifovea, the varying widths of the areas of interest (3 x 3 mm^2^ or 6 x 6 mm^2^) and the use of different methods for comparison of the VDs (comparing total VD, foveal VD, parafoveal VD, perifoveal VD, or comparing the VDs quadrant by quadrant) among the studies might have contributed to divergent results.

As the exact pathophysiological mechanism of the retinochoroidal microvascular alterations caused by HM is yet to be understood, the preserved FAZ area and increased foveal VD in the SCP and DCP segments observed in the present study could be attributed to the possible preservation of the FAZ area through autoregulatory mechanisms, associated with its high metabolic activity and primary role in central vision [8]. Even though it has been previously speculated that biomechanical stretching of the retina, choroid, and sclera due to axial elongation can cause straightening and narrowing of the vessels, leading to a reduction in capillary density [37], the inclusion of eyes with degenerative changes in this study could be another possible explanation for the increased VD in the fovea. Our results further indicated that mechanical stretching might not be the only factor contributing to the microvascular alterations observed in HM. This suggests the involvement of additional mechanisms beyond biomechanical forces in the development of these microvascular changes.

Histopathological examinations have indicated that the choroidal thinning associated with the axial elongation specifically impacted the medium to large choroidal vessels. At the same time, the thickness of the CC showed no correlation with AL in myopic patients [41,42]. In addition, Cheng et al. [21] found no significant changes in foveal and parafoveal CC perfusion between the myopic and non-myopic eyes. Jiang et al. [7] highlighted the similarity in choroidal VD between myopic and non-myopic eyes. Although the present study did not include an emmetropic control group, we observed a decrease in total VD and VDs in the CC segment in the temporal, inferior, and nasal parafoveal quadrants with an increase in AL.

While the OCTA findings in the SCP, DCP, and CC segments have been frequently examined in high myopic eyes, very few publications report data on the OuR segment. Meng et al. [6] emphasized there was even a statistically significant decrease in the average VD values in both the SCP and DCP segments, along with a reduction in choroidal blood flow in the CC segment with an increase in the degree of myopia, with no significant relationship between the degree of myopia and the blood flow in the OuR. The present study demonstrated an increase in the foveal VD and a decrease in the superior parafoveal VD in the OuR segment with the rise in AL. The increase in foveal VD in the OuR segment could have resulted from including eyes with active MNV.

In most previous studies on HM, sample sizes were limited, different definitions of HM were used, numerous parameters were examined, and, more importantly, measurements were taken with various OCTA devices, which prevented meaningful comparisons among the studies. In contrast, our research brought a different perspective by including patients with a wide age range and also eyes exhibiting myopic degenerative findings, such as myopic maculopathy equal to or higher than META-PM category 2, MNV, and/or PS, in an attempt to provide a broad representation of high myopic eyes. This approach aimed to provide a comprehensive representation of high myopic eyes. However, it is essential to recognize that this inclusion might have also limited our study, as these degenerative changes could have potentially influenced the results. This study’s retrospective, single-center design, coupled with a relatively small sample size and manual measurement of FAZ areas, represents additional limitations that must be acknowledged. Furthermore, the lack of differences in this clinical population may not accurately reflect the entire high myopic population worldwide due to the study's monoracial background. In the future, OCTA studies examining a substantially larger cohort of eyes from a more heterogeneous population with varying severities of HM prospectively and longitudinally will likely yield new insights into the pathophysiology of HM and its associated complications, thereby enhancing our understanding of the disease.

## Conclusion

In conclusion, our OCTA findings reflected some new insights into high myopic eyes. We revealed that high myopic eyes with longer ALs exhibited an increased total VD in the DCP and increased foveal VD in the SCP, DCP, and OuR segments. In contrast, those eyes showed decreased total VD and temporal, inferior, and nasal parafoveal VDs in the CC segment compared to high myopic eyes with shorter ALs. Our OCTA analysis extends the common understanding of HM-related microvascular changes. Additional studies seem necessary to determine the exact pathophysiology and clinical relevance of the microvascular changes in eyes with HM.
